# COVID-19-related stigma within a rural South African community: A mixed methods analysis

**DOI:** 10.1371/journal.pone.0306821

**Published:** 2024-07-18

**Authors:** Duduzile P. Mashinini, Nicole K. Kelly, Palesa Mataboge, Frantasia Hill, Harish Nair, George Palattiyil, Kathleen Kahn, Audrey Pettifor

**Affiliations:** 1 Carolina Population Center, Biosocial Training Program, The University of North Carolina at Chapel Hill, Chapel Hill, North Carolina, United States of America; 2 Department of Epidemiology, Gillings School of Global Public Health, The University of North Carolina at Chapel Hill, Chapel Hill, North Carolina, United States of America; 3 MRC/Wits Rural Public Health Transitions Research Unit, School of Public Health, Faculty of Health Science University of the Witwatersrand, Johannesburg, South Africa; 4 Centre for Global Health, Usher Institute, University of Edinburgh, Edinburgh, United Kingdom; 5 Social Work, School of Social and Political Science, University of Edinburgh, Edinburgh, United Kingdom; 6 Department of Social Work and Community Development, University of Johannesburg, Johannesburg, South Africa; University of KwaZulu-Natal College of Health Sciences, SOUTH AFRICA

## Abstract

**Background:**

Infectious disease-related stigma is a pervasive global issue that impedes disease control efforts by increasing reluctance to seek treatment or engagement in prevention behaviors for fear of ostracism. Despite this, there is limited research on COVID-19 stigma in Africa, specifically rural South Africa, which has faced infectious disease-related stigma throughout the HIV epidemic.

**Methods:**

Population-based surveys were conducted among 1,662 adults living in the Agincourt Health and Socio-Demographic Surveillance System (AHDSS) area in Mpumalanga, South Africa, in August-October 2020 and August-October 2021. Surveys measured anticipated COVID-19-related stigma from low to high levels. Changes in stigma between surveys were compared using Wilcoxon ranked sign tests, and log-binomial models estimated the association between socio-demographic factors and anticipated stigma at both intervals. Qualitative interviews were conducted in 2022 among 31 adults who completed the original surveys, and the data were analyzed thematically to describe anticipated, perceived, and enacted stigma.

**Results:**

Anticipated stigma significantly decreased from the first to the second survey (p-value:<0.0001). Stigma was significantly higher among older age groups. In 2020, those less knowledgeable about COVID-19 were 2.24 times as likely to have higher levels of anticipated stigma compared to those who were more knowledgeable (RR:2.24, 95% CI: 1.90,2.64). Fear of being stigmatized influenced willingness to disclose infection. Participants perceived COVID-19 stigma as similar to HIV/AIDS stigma, but concern and fear reduced over time, with differences observed across generations and sexes. For some, fear of death and mistrust of others endorsed enacting stigma toward others.

**Conclusion:**

While COVID-19 stigma decreased over time in rural South Africa, different forms of stigma persisted and influenced participants’ willingness to reveal their COVID-19 infection status. Given South Africa’s history with infectious disease-related stigma hindering public health efforts, it is crucial that government bodies prioritize strategies to mitigate stigma in rural communities.

## Introduction

The WHO defines disease-related stigma as “the negative association between a person or group of people who share certain characteristics and a specific disease [1].” During a pandemic such as COVID-19, this stigma may manifest as harmful stereotypes, unfair treatment, and decreased social status due to a perceived association with a particular disease [2]. Infectious disease-related stigma, primarily informed by HIV stigma theory [2], is known to occur in many forms (perceived, enacted, anticipated, and internalized stigma) and at many levels (individual, community, and structural levels) [2, 3]. Regardless of the stigmatized disease of interest, researchers have identified stigma as a critical population-level driver of morbidity and mortality and a fundamental cause of health inequities [3]. In the context of COVID-19, a growing body of evidence implicates stigma as a significant barrier to testing and other protective health behaviors [4, 5]. For instance, in their study assessing anticipated stigma, stereotypes, and COVID-19 testing among participants in the United States, Earnshaw and colleagues found that those who anticipated COVID-19-related stigma to a greater degree reported being less likely to seek a COVID-19 test [4].

In South Africa, a country with the most people living with HIV globally [6], and where stigma has played a substantial role in undermining HIV prevention and treatment efforts, [7, 8] COVID-19-related stigma has also undermined COVID-19 control efforts [5–9]. In an attempt to address COVID-19-related stigma, during a June 2020 presidential address, South African President Cyril Ramaphosa issued a familiar refrain calling for not only the “collective responsibility to stamp out stigmatization, but also understanding, tolerance, kindness, empathy, and compassion for those who are infected with [COVID-19] and for their families [10].”

Although infectious disease-related stigma, including COVID-19-related stigma, is an issue many communities face, rural communities are particularly vulnerable to its undesired social and health consequences, as they often lack anonymity and sufficient access to mental health resources [11]. This increased vulnerability to disease-related stigma in rural areas is well documented [12]; however, there is insufficient research on COVID-19-related stigma in rural communities, particularly in South Africa, the African nation with the greatest COVID-19 morbidity and mortality burden [13]. Beyond COVID-19, South Africans have witnessed infectious disease-related stigma throughout the nation’s long-standing HIV epidemic [14–16]. Now, with the endemicity of the global coronavirus pandemic, discrimination toward those infected with COVID-19 has been reported [17]. Having suffered decades of HIV-related stigma, South Africa is a unique setting to study COVID-19-related stigma. Therefore, this study aimed to investigate anticipated COVID-19-related stigma quantitatively and, secondly, to explore anticipated, enacted, and perceived COVID-19-related stigma qualitatively in rural Mpumalanga, South Africa, from 2020–2022.

## Methods

We conducted a mixed methods study to measure the social, economic, and behavioral impacts of COVID-19 from 2020–2022. The study assessed COVID-19-related stigma among adults aged 18 and older residing within the Agincourt Health and Socio-Demographic Surveillance System (AHDSS) study area. We used qualitative and quantitative methods to measure anticipated, enacted, and perceived COVID-19-related stigma among participants. In the context of this study, anticipated stigma refers to the expectation of stigma in the future [4], enacted stigma includes discriminatory acts and behaviors [18], and perceived stigma is a person’s understanding of how others may act towards, think, and feel about them if they were to contract COVID-19 [18].

For the quantitative part of the study, participants provided verbal informed consent, which was recorded by the call center and documented by fieldworkers using a questionnaire. For the qualitative component, participants completed physical consent forms before beginning the study. The study gained written approval from the Human Research Ethics Committee at the University of Witwatersrand (number: M190305) and the University of North Carolina’s Institutional Review Board (number: 21–0011). Details on the study design for the quantitative and qualitative components can be found in their respective sub-sections (below).

### Study setting

All data were collected within the AHDSS study area [19, 20], located in a rural and low-resource area of Mpumalanga province, northeast South Africa, near the border with Mozambique. The study area is characterized by high unemployment with consequent high labor migration rates of men and, increasingly, women. There is a high reliance on government social support grants, poor quality education, and low literacy of older adults. This area also has poor water and sanitation, with most villagers reliant on water from communal standpipes. The AHDSS was established in 1992, and its study area covers a population of some 117,000 persons in 20,000 households in 31contiguous villages. The AHDSS seeks to acquire information on births, deaths, and migrations collected annually through an in-person household visit; since 2020, two telephonic visits have been added annually. Additional data collection modules, such as on labor migration, are collected regularly but less frequently [20].

### Quantitative study

#### Study population and design

A longitudinal, population-based cohort study was conducted among adults living in the AHDSS region during the COVID-19 pandemic. This was a separate cohort study that consisted of two repeated telephonic surveys rather than using existing AHDSS data. The first round of data collection occurred early in the pandemic (August-October 2020) when less was known about the virus and before any COVID-19 vaccine approvals. The follow-up survey was conducted one year later (August-October 2021) after several vaccines had been approved but were not widely available in rural South Africa. Vaccine eligibility also changed during the second round of data collection: COVID-19 vaccines (Pfizer and Jansen) became available for South Africans aged 60 and older in May 2021 and were available for those 18 and older beginning in September 2021 [21]. All surveys were conducted in the local language (Shangaan) and were translated and back-translated from English.

Participants were eligible to participate in the study if they were 18–79 years of age and were permanently residing within the AHDSS. The sampling frame for this study was the AHDSS. To ensure that the study was representative of the 2020 ADHSS with respect to sex and age, we stratified the sample by biological sex (female or male) and by age group (18–29, 30–39, 40–49, 50–49, 60–69, 70+). We then randomly sampled individuals within each combined sex and age stratum, resulting in a study sample of 2,300 individuals (one individual per ADHSS household). 2,223 of these 2,300 participants completed the first telephonic survey (97%); of those, 1,664 completed the second survey a year later (75%). For the second survey, non-responses were due to failed calls/ invalid numbers (N = 59), call back later (N = 29), callbacks that went to voicemail (N = 178), refusals (N = 72), wrong numbers (N = 124), no answers (N = 16), incomplete interviews (N = 37), and deceased participants (N = 8).

Only participants who completed both surveys and had available COVID-19 vaccination information at the follow-up survey (N = 1,662) were included in this analysis. To further ensure balance, we re-weighted the final analytic sample (N = 1,662) by age and sex to the 2020 ADHSS, resulting in a final weighted sample size of 34,582 individuals.

#### Measures

In both surveys, participants were asked four questions on a 3-point Likert scale about anticipated COVID-19-related stigma: how likely they thought the following would happen if they tested positive for COVID-19 (very likely, somewhat likely, or unlikely): 1) lose their friends, 2) be disowned or neglected by their family, 3) be treated very badly by health professionals, and 4) be treated like a social outcast by their community. These questions were adapted from existing anticipated HIV stigma scales [22, 23] to be relevant in the context of COVID-19. A composite stigma score was created by summing responses to the four questions (minimum score = 4 points (low stigma); maximum score = 12 points (high stigma)). A binary stigma indicator was operationalized (high stigma (index) = highest quartile of the composite stigma score; low stigma (reference group) = combined three lowest quartiles of the stigma score).

During the first survey in 2020, eight true/false questions were asked about COVID-19 to gauge general knowledge, given the emerging nature of the disease. Questions about COVID-19 symptoms, transmission, prevention, and commonly spread misinformation were asked. An overall knowledge score was created by summing the number of correct responses (0 = least COVID-19 knowledge; 8 = most COVID-19 knowledge). A binary indicator of COVID-19 knowledge was created from this score (less knowledgeable (index) = lowest quartile of knowledge scores; more knowledgeable (reference group) = combined three highest quartiles of knowledge scores). Additional variables of interest included age group (ascertained at round 1), sex (ascertained at round 1), and COVID-19 vaccination status (only measured in the follow-up survey), disclosure of COVID-19 vaccination status to friends/family (for those vaccinated), and perceived likelihood of developing illness from COVID-19 infection; all were self-reported.

#### Statistical analysis

Descriptive statistics of anticipated COVID-19-related stigma are presented for each time point. Wilcoxon ranked sign tests were used to compare changes in stigma scores between the first and second surveys, given the non-normal distributions. Log-binomial models were used to estimate risk ratios (RRs) and 95% confidence intervals (CIs) for the association between sociodemographic factors (age, sex, COVID-19 vaccination status, COVID-19 knowledge) and COVID-19-related stigma. Covariate adjustment sets for the regression models were identified from directed acyclic graphs [24] and available survey information: models for age and sex did not adjust for any covariates, and models for vaccination status and COVID-19 vaccination status were controlled for age and sex. Cronbach’s alphas are reported for the COVID-19-related stigma scale at each time point.

Inverse probability of sampling weights were applied to the final analytical sample (N = 1,662) to ensure that it was representative of the 2020 AHDSS with respect to age and sex (weighted AHDSS population = 34,582). Thus, the quantitative results presented in this paper are all weighted values, including counts, percentages, measures of association, and 95% CIs. All statistical analyses were two-sided (alpha = 0.05) and were conducted using Stata version 17.0 (StataCorp LLC, College Station, Texas, USA). Density plots were generated using R version 4.2.0 (R Foundation for Statistical Computing).

#### Qualitative study

To assess COVID-19-related stigma from the participant’s perspective, in-depth face-to-face interviews (IDIs) were conducted one time with a sample of participants from the second survey. Participants were purposefully sampled for the IDIs using the respondent’s self-reported vaccination status derived from the telephonic survey question, “Have you received a COVID-19 vaccine?” We sampled participants for IDIs until we reached data saturation. This involved randomly selecting 16 individuals who had been vaccinated to form the vaccinated group and randomly choosing 15 individuals who indicated they would definitely not get vaccinated against COVID-19 to form the unvaccinated group, totaling 31 participants. Interviews were conducted from January to February 2022.

Interviews were conducted in participants’ homes for roughly 30 minutes each. Interviews were implemented by trained staff in the participant’s language and transcribed into English verbatim. All interviewers were trained local female residents of the 31 contiguous villages where the study was conducted. Interviewers took field notes during the interview, and when transcript clarification was needed, they reconnected with participants for clarification about their answers. Following transcription, a thematic content analytical approach was undertaken, whereby participants’ accounts were analyzed for patterns leading to code construction, ultimately forming themes [25]. Codes were generated using the interview guide, and participants’ transcripts were reviewed to identify recurring views, experiences, and beliefs. Responses were then labeled and organized into categories to form the themes. Dedoose qualitative software version 9.0.54 (SocioCultural Research Consultants, LLC, Los Angeles, California, USA) was used to organize qualitative data, construct a codebook, and systematically apply codes to the transcripts. Codes were assessed for inter-rater reliability among four authors, achieving Cohen’s Kappa > .74.

## Results

### Quantitative results

#### Demographic characteristics

Of the 1,662 participants in this population-based cohort study in 2020 (corresponding to a weighted sample of 34,582 individuals), the majority were female (59.8%), relatively young (largest age group: 18–29-year-olds (39.2%)), and unemployed (76.6%) at the 2020 baseline survey (Table 1). One-third of participants reported household food insecurity in the past month, and few participants self-reported any previous COVID-19 infections (1.2%, Table 1).

**Table 1:** Demographic characteristics among a representative sample of adults residing within the Agincourt Health and Socio-demographic Surveillance System from August-October 2020 in August-October 2020 and August-October 2021.

	Round 1		Round 2	
	(Aug-Oct 2021)		(Aug-Oct 2021)	
	N	%	N	%
	(Total = 34,582)	(95% CI)	(Total = 34,582)	(95% CI)
**Sex**				
Male	13,909	40.2 (37.8,42.7)	13,909	40.2 (37.8,42.7)
Female	20,673	59.8 (57.3,62.2)	20,673	59.8 (57.3,62.2)
Missing	0	0	0	0
**Age group**				
18–29	13,548	39.2 (36.7,41.7)	12,711	36.8 (34.3,39.3)
30–39	7,115	20.6 (18.7,22.6)	7,351	21.3 (19.4,23.3)
40–49	5,396	15.6 (14.0,17.3)	5,576	16.1 (14.5,17.8)
50–59	4,201	12.1 (10.7,13.7)	4,283	12.4 (10.9,14.0)
60–69	2,868	8.3 (7.1,9.6)	3,011	8.7 (7.5,10.1)
70+	1,454	4.2 (3.4,5.2)	1,650	4.8 (3.9,5.8)
Missing	0	0	0	0
Employment status^1^				
Yes	8,095	23.4 (21.4,25.5)	10,998	31.8 (29.5,34.2)
No	26,487	76.6 (74.5,78.6)	23,172	67.0 (64.6,69.3)
Missing	0	0	412	1.2 (0.8,1.8)
Food insecurity^2^				
Yes	11,552	33.4 (31.1,35.8)	11,172	32.3 (30.1,34.6)
No	23,030	66.6 (64.2,68.9)	23,016	66.6 (64.2,68.8)
Missing	0	0	394	1.1 (0.7,1.8)
Previously infected with COVID-19^3^				
Yes, laboratory confirmed	423	1.2 (0.8,1.9)	865	2.5 (1.8,3.4)
Symptomatic, not laboratory confirmed	0	0	262	0.8 (0.4,1.4)
No, never infected	33,887	98.0 (97.2,98.6)	33,322	96.4 (95.3,97.2)
Missing	272	0.8 (0.5,1.4)	133	0.4 (0.2,0.8)

^1^Self-reported any work for pay

^2^In the past month having no food at all in your household, going to bed hungry, or going a whole day without eating

^3^Self report

#### Anticipated COVID-19-related stigma

From August-October 2020, two-thirds of participants indicated that they would be very likely or somewhat likely to lose friends if they became infected with COVID-19 (Table 2). However, this number decreased to less than half of the participants when measured one year later (August-October 2021). In 2020, 24.9% thought that it was somewhat or very likely that they would be neglected or disowned by their family for contracting COVID-19 (Table 2). This number decreased to 12.1% one year later. Almost half of the participants in 2020 anticipated that they would be treated poorly by health professionals if they became infected with COVID-19, but this percentage decreased to 36.8% in 2021. Initially, 71.5% of participants indicated that their community would treat them like outcasts if they contracted COVID-19; however, this number decreased to 46.3% one year later.

**Table 2:** Anticipated COVID-19-related stigma among a representative sample of adults residing within the AHDSS^1^ from Aug-Oct. 2020 and Aug.-Oct. 2021.

	August-October 2020 (N = 34,582)		August-October 2021 (N = 34,582)	
	N	% (CI)	N	% (CI)
***COVID-19-related Stigma*: *How likely are you to…***				
**Lose your friends**				
** **Very likely	12,734	36.5 (34.1,38.9)	5,859	16.9 (15.2,18.9)
** **Somewhat likely	10,493	30.0 (27.8,32.3)	9,293	26.9 (24.8,29.1)
** **Unlikely	12,734	33.3 (31.0,35.7)	19,169	55.4 (53.0,57.9)
** **Missing	71	0.2 (0.1,0.7)	261	0.8 (0.4,1.3)
**Be neglected/disowned by your family**				
** **Very likely	4,130	11.8 (10.3,13.4)	1,357	3.9 (3.1,5.0)
** **Somewhat likely	4,577	13.1 (11.5,14.8)	2,837	8.2 (7.0,9.6)
** **Unlikely	26,223	75.0 (72.9,77.1)	30,253	87.5 (85.8,89.0)
** **Missing	38	0.1 (0.0,0.4)	135	0.4 (0.2,0.8)
**Be treated poorly by health professionals**				
** **Very likely	6,293	18.0 (16.2,20.0)	2,472	7.1 (6.0,8.5)
** **Somewhat likely	10,520	30.0 (27.8,32.4)	10,260	29.7 (27.5,32.0)
** **Unlikely	18,081	51.7 (49.3,54.2)	21,524	62.2 (59.8,64.6)
** **Missing	74	0.2 (0.1,0.6)	326	0.9 (0.6,1.5)
**Be treated like a social outcast by your community**				
** **Very likely	14,207	40.7 (38.3,43.1)	5,295	15.3 (13.6,17.2)
** **Somewhat likely	10,815	30.8 (28.6,33.1)	10,709	31.0 (28.7,33.3)
** **Unlikely	9,768	28.0 (25.8,30.3)	18,199	52.6 (50.2,55.1)
** **Missing	177	0.5 (0.3,1.0)	379	1.1 (0.7,1.7)
**COVID-19-related stigma score**^**2**^: Median (IQR)	7	(5,9)	6	(4,7)
**COVID-19 Knowledge score**^**3**^: Median (IQR)	7	(6,8)	---	---
**Received COVID-19 vaccine**	---	---	17,424	50.4 (47.9,52.9)
**Disclosed COVID-19 vaccine status**	---	---	16,595	95.2 (93.6,96.5)
**Concern that you will become seriously ill from COVID-19 in the next 3 months**				
** **Not at all	9,732	28.1 (26.0,30.4)	14,251	41.2 (38.8,43.7)
** **Slightly	4,540	13.1 (11.6,14.9)	5,635	16.3 (14.5,18.2)
** **Moderately	4,769	13.8 (12.2,15.6)	5,825	16.8 (15.1,18.8)
** **Extremely	15,525	44.9 (42.4,47.4)	8,872	25.7 (23.5,27.9)
** **Missing	16	0.0 (0.0,0.3)	0	---

^1^Agincourt Health and Socio-demographic Surveillance System

^2^Stigma scores were calculated by summing the responses to the 4 anticipated stigma questions on a 3-point Likert scale. Higher stigma scores correspond to higher levels of anticipated COVID-19 stigma (possible range: 4,12)

^3^COVID-19 knowledge scores were based on the number of 8 COVID-related questions that were answered correctly (highest possible score = 8)

**Fig 1:**
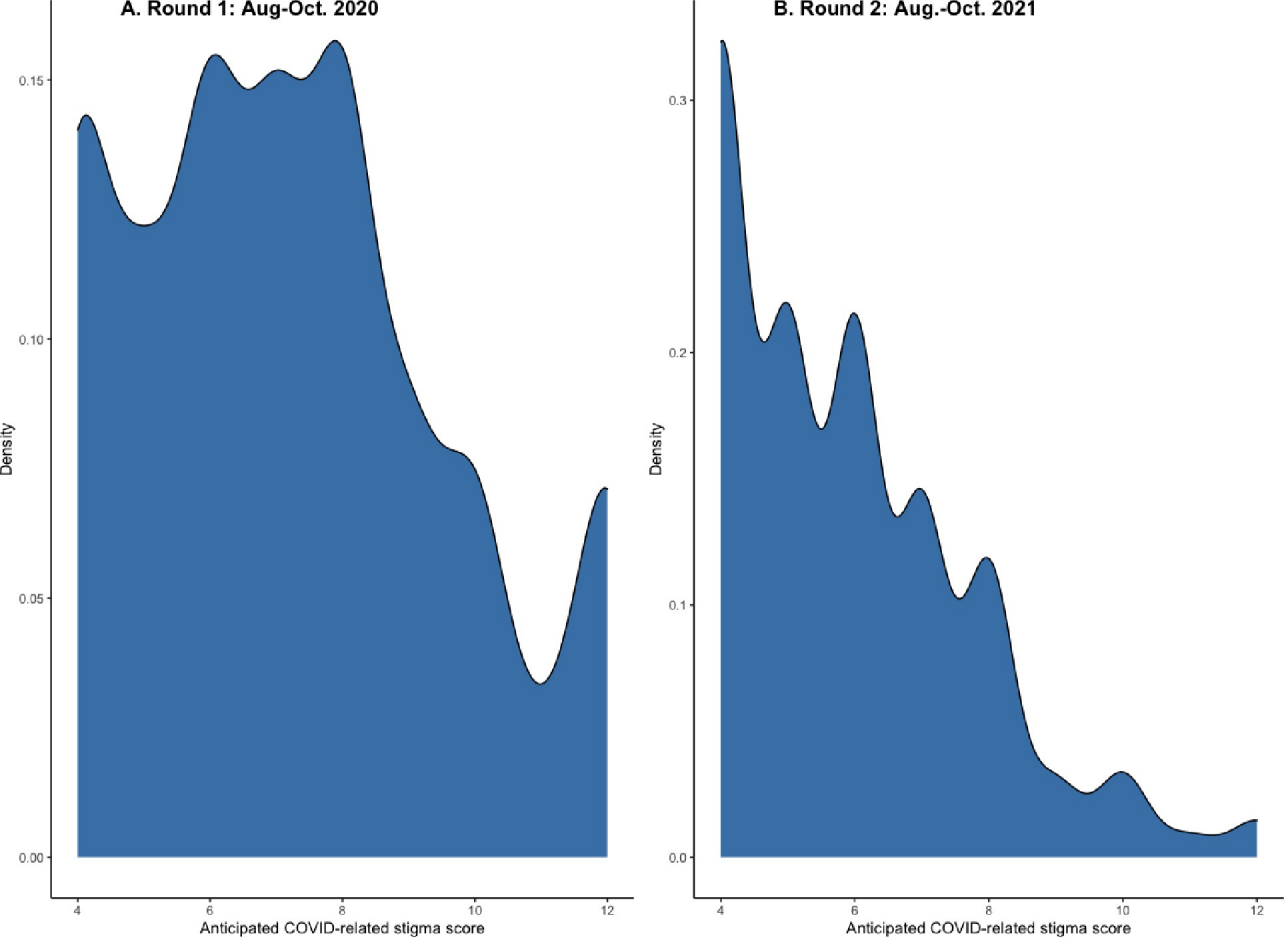
Distribution of COVID-19 anticipated stigma in Aug-Oct 2020 and Aug-Oct 2021.

In 2020, the median COVID-19-related stigma summary score was 7, with an interquartile range (IQR) of 5,9 (Table 2). By 2021, the median score had significantly decreased to 6 (IQR: 4,7, Wilcoxon p-value: <0.0001) (Tables 2 & 3; Fig 1). The median COVID-19 knowledge score (measured only in 2020) was 7 out of 8 points (IQR: 6,8) (Table 2). Cronbach’s alpha for the COVID-19-related stigma scale was 0.76 at baseline and 0.64 for the follow-up survey. Concern about COVID-19 infection decreased substantially during the study. At baseline, 44.9% were extremely concerned that they would become seriously ill from COVID-19, compared to 25.7% one year later (Table 2).

**Table 3:** Associations between sociodemographic factors and anticipated COVID-19-related stigma from Aug-Oct 2020 and Aug-Oct 2021.

	August-October 2020		August-October 2021	
	Risk ratio	95% CI	Risk ratio	95% CI
***COVID-19-related Stigma*:**				
**Sex**				
** **Female	1.11	0.93,1.33	1.15	0.98,1.35
**Age**				
** **30–39	1.10	0.86,1.41	1.20	0.96,1.51
** **40–49	1.43	1.13,1.80	1.42	1.14,1.76
** **50–59	1.07	0.81,1.42	1.47	1.16,1.87
** **60–69	1.41	1.07,1.86	1.65	1.30,2.11
** **70+	0.99	0.66,1.48	1.17	0.82,1.68
**COVID-19 vaccination status** ^**2**^				
** **Unvaccinated	---	----	1.14	0.97,1.32
**COVID-19 knowledge** ^**3**^				
** **Less knowledgeable of COVID-19	2.24	1.90,2.64	---	---
**Change in COVID-19-related stigma** ^**4**^	Wilcoxon p-value: < 0.0001			

^1^Models for sex and age were unadjusted; COVID-19 vaccination status and knowledge models controlled for age and sex; covariates were selected based on directed acyclic graphs and available information

^2^Vaccination status was only ascertained in Round 2, as COVID-19 vaccines were not available during Round 1

^3^COVID-19 knowledge was only collected in Round 1

^4^Changes in anticipated COVID-19-related stigma from 2020 to 2021 were compared using Wilcoxon Signed Rank tests

#### Associations between sociodemographic factors and COVID-19-related stigma

Women had slightly higher levels of anticipated COVID-19-related stigma than men, although this was not statistically significant at either time point (2020 risk ratio: 1.11, 95% CI: 0.93,1.33; 2021 RR: 1.15, 95% CI: 0.98,1.35, Table 3). Anticipated COVID-19-related stigma did vary by age (Table 3). In 2020, 40–49-year-olds and 60-69-year-olds had significantly higher anticipated COVID-19-related stigma levels compared to 18-29-year-olds (RR: 1.43, 95% CI: 1.13,1.80; RR: 1.41, 95% CI: 1.07,1.86, respectively); however, this association was not present among other age groups. In 2021, we observed higher stigma levels among all age groups compared to 18-29-year-olds, with the exception of those ages 70 and older (although not statistically significant among 30-39-year-olds) (Table 3). At the second study visit, half of the survey population reported receiving a COVID-19 vaccine (50.4%, 95% CI: 47.9, 52.9), and of those vaccinated, almost all had disclosed their COVID-19 status to a friend or family member (95.2%, 95% CI: 93.6, 96.5) (Table 2). COVID-19 vaccination status was not significantly associated with a higher risk of COVID-19-related stigma; however, the findings may indicate a preliminary signal that unvaccinated individuals reported higher levels of stigma (RR: 1.14, 95% CI: 0.97,1.32; Table 3). The risk of anticipating COVID-19-related stigma in 2020 was higher among those who were less knowledgeable about the disease (RR: 2.24, 95% CI: 1.90, 2.64; Table 3).

### Qualitative results

The study’s IDIs revealed six themes of COVID-19-related stigma. The first illustrated how participants’ anticipation of stigma led to hiding COVID-19 infection status and distancing from COVID-19, as evidenced by how some spoke about the disease. However, a greater level of COVID-19 knowledge mitigated the impact of anticipated stigma for some, making them more willing to share their infection status. The second theme compared COVID-19 to HIV/AIDS and portrayed participants’ anticipation and perception of COVID-19-related stigma, drawing similarities to HIV/AIDS-related stigma. While interrelated, the third and fourth themes, fear of death and decreased COVID-19 concern, were documented separately due to the juxtaposition in attitude. However, the two themes were similar in revealing sex and generational differences and, in some instances, warranted participants’ enactment of stigma against others. The fifth theme, mistrust of others, also justified some participants’ enactment of stigma towards others who were thought to come from high-risk areas. However, some indicated that this was motivated by the perception that others had malicious intent to spread COVID-19 purposely. The final theme showcased the anticipation of being discriminated against for disclosing COVID-19 vaccination status and how some participants enacted stigma towards vaccinated individuals (Table 4).

**Table 4:** COVID-19-related stigma themes, associated stigma types, and quotes from adults in rural South Africa.

Themes	Sub-theme	Stigma Type	Participant Quotes
**Hiding infection status & COVID distancing**	**Distancing**	**Anticipated**	**Question**: **Do you think people feel comfortable speaking to others about having had COVID-19?HOBQT** P: No, because people are ashamed of sharing if they have COVID-19, so they hide it from others. I: Is this something people should hide from others? P: People do hide from others, because when you have COVID-19 you have to isolate yourself and quarantine, and people don’t want that. **DQQOS** P: No, they are uncomfortable**Question**: **What are your experiences with COVID-19?HKCRJ** P: I don’t know anything; I have just heard that there is COVID-19.
**Comparing COVID-19 to HIV**	**Sex**	**Anticipated & Perceived**	**Question****: Do people hide that they are infected or were infected with COVID-19?** **HKCRJ** P: This is not something you should hide because you didn’t get it by sleeping with a man… **BSRQP** P: It’s not a good thing for people not to share because, if people would share if they have COVID-19, maybe people would help them. Just like HIV, people used to hide it and a lot of people have died even now people are still doing so. P: …the sad part about COVID-19 is that it is unlike HIV, because a person would only be infected with HIV because of being engaged on sexual intercourse, so with COVID-19, you don’t engage on sexual intercourse, you are always indoors but at the end of the day you will have COVID-19 **EGHCC** P: Males don’t take COVID-19 and HIV seriously.
**Fear of death**	**Age**	**Enacted**	**Question:** **What is the reason why people don’t share with others if they have COVID-19?** **BSRQP** P: People don’t share with people that they have COVID-19 because, they feel like people will not want to be around them because COVID-19 kills. **Question:** **How concerned are people in your community about COVID-19?** **HKCRJ** P: …this thing is not good, I was chatting on WhatsApp this other day and I saw that one of my siblings is infected with COVID-19, I was not happy about the situation because she looked sick, so it shows that this virus is bad and it kills… This thing is scary because it kills and if it spreads it can kill all of us. P: Old people are the ones who follow the regulations. I: Why do you say that it’s old people? P: They are afraid of death; they were told that if they get infected, they will die. **Question: Can you tell me what you know about Covid-19?** **GEMVF** P: Covid-19 is a disease which kills
**Decreased COVID-19 concern**	**Age**	**Enacted**	**Question:** **How concerned are people in your community about COVID-19?** **HNRZT** P: They were concerned at first but now they are no longer concerned. **HQTDN** P: …older people are still worried because they are the ones who are in a greater risk. **CGQOK** P: …they [males] don’t trust that COVID-19 is real, they want to believe it when someone close to them died[s].
	**Sex**		
**Mistrust & fear of contact with others**		**Enacted**	**Question:** **Do people feel comfortable talking to others about having had COVID-19?** **CQRRY** P: They don’t care about other people; they won’t tell them if whether they have it or they once had it they want people to find out on their own. **Question: What gatherings do people attend despite being afraid of Covid-19?** **HKCRJ** **P:** [Funerals]. Family members are the ones that are supposed to attend, but because people are disrespectful, they still go there and get overcrowded. The sad part about this is that some family members come from different areas like Gauteng and that’s where there is a high number of people who are infected with Covid-19. P: There was a funeral in our area, and they came to visit the family of the deceased one of the people was infected with Covid-19, that person infected the parents of the deceased and they both died. **Question: Do you think going out increases the risks of getting Covid 19?** **LLPGO** P: yes I: why do you think so? P: because we will be infected as there will be lots of people… Others might go because they want to spread Covid 19 intentionally.
**COVID-19 vaccine related stigma**		**Anticipated**	**Question:** **What makes others not to talk about their vaccine status?** **EFDMV** P: I have heard them [people] saying that this vaccine thing is written even in the bible and that it is satanic. **CBKJT** P: Some people are still saying that it’s satanic, you will die after getting vaccinated and that you won’t have children, but others believe that it works. **EHLFS** P: They said that it kills many people after that was said, they were afraid to go and vaccinate.

#### Hiding infection status & COVID-19 distancing

Anticipated COVID-19-related stigma was expressed in participants’ unwillingness to disclose COVID-19 infection status to others, as participants spoke about being infected with COVID-19 as a source of shame worthy of hiding (Table 4). When asked if people feel comfortable talking to others about having COVID-19, one participant said, *“… if you tell them [people] that you have COVID or you once had it*, *they will be afraid to get in contact with you*.*”* Most participants did not feel that sharing one’s infection status was welcomed or supported by others, leading to participants seeming to distance themselves from COVID-19 (Table 4). When asked, “What are your experiences with COVID-19?” one participant responded, “*I never experienced COVID-19; I only see it on TV*.”

Echoing people’s desire to hide their COVID-19 status for fear of stigma even after recovering from the disease, a participant proposed, “*They are afraid of being stigmatized*, *and people might think they still have it even after full recovery*.” Emphasizing their fear of being stigmatized, a participant said, “*There’s no such thing as talking freely of having COVID-19*.” However, for some, possessing a high level of COVID-19 knowledge seemed to reduce fear of disclosure, “*For someone like me*, *I’d say I would feel comfortable talking because I have learned so much about COVID-19…”* These findings support the high levels of anticipated stigma reported by participants in the quantitative survey in which the level of COVID-19 knowledge was tied to a decrease in anticipated stigma and the likelihood of being seen as an outcast of the community, family, and friends.

#### Comparing COVID-19 to HIV/AIDS

Participants anticipated and perceived COVID-19 stigma to be similar to HIV/AIDS stigma, as they consistently compared COVID-19 stigma to HIV/AIDS stigma in a way that reflected their expectations and experiences of stigma (Table 4). When probed to answer why people may hide their COVID-19 infection status, a participant drew from South Africa’s experiences with the HIV/AIDS epidemic, saying, “…*When you look back where we are coming from*, *where a person was infected with HIV/AIDS*, *we couldn’t tell even our partners that we are HIV positive*.” Akin to this, another participant spoke of perceiving COVID-19-related stigma as parallel to omnipresent HIV/AIDS stigma, stating, “…*Maybe they are afraid of stigma*, *just like how people* [are] *stigmatizing people living with HIV*. *People living with HIV don’t disclose their HIV status; they even choose to take it to their graves*…*”* Documenting perceived changes in the severity of HIV infection since the start of the HIV pandemic, one participant stated, “*COVID-19 kills; it is unlike HIV because with HIV*, *you will take medication and be fine*, *but COVID-19 kills*.”

#### Fear of death

In several instances, COVID-19-related stigma was strongly linked to the fear of death and, for some, served as a pretext for enacting stigma against others (Table 4). For instance, a participant spoke of avoiding funerals where the deceased had COVID-19, saying, “…[If] *I do not know the cause of death*, *I do not attend because COVID-19 doesn’t take time to kill a person*, *you die fast*.” Similarly, when talking about attending funerals where some family members were traveling from Gauteng, a province perceived to be a high COVID-19 prevalence area, a participant talked about how sad it was that she was discriminating against those from these areas, stating, “*We are scared to go to such places because we meet different people from different environment*.”

#### Differences in fear by sex & age

Fear of death from COVID-19 for some was qualitatively associated with sex and age, where women and older people were perceived to be more concerned about COVID-19 and more likely to take precautionary measures such as wearing masks and getting vaccinated (Table 4). When asked about the perception of the level of COVID-19 concern in the community, a participant spoke to the perceived sex differences, saying, **“***Mostly people who fear COVID-19 are females; males don’t care about this pandemic because they are always at taverns where they are many…At funerals*, *you will find most females wearing masks*.” Speaking about the generational differences, when asked to talk about community COVID-19 concerns, a participant said they thought, “*Old people are more scared about COVID-19 than young people*.” In agreement with the notion of generational differences and speaking to the sustained level of concern and fear, an older participant confirmed this, stating, “*It’s us older people who are very much concerned because they said that* [it] *affects people who* [are] *60 years* [and] *upwards*, *we are really sad because of that we think that we are going to die*.”

#### Decreased COVID-19 concerns

Many participants discussed the decreased COVID-19 concern at the individual and community levels as the pandemic wore on (Table 4). Some attributed the change to habituation, or as one participant put it, “*We have learned to live with COVID*.” Others attributed the decreased concern in the community to the availability of vaccines and the perception that not as many people were dying from COVID-19: “*Now that they have introduced the vaccine*, *they no longer care much about COVID-19 because they know that there’s a cure*.” However, while some participants welcomed the decrease in fear and anxiety, others perceived it as premature and irresponsible. These conflicting perspectives engendered tensions, leading some to enact stigma toward others they perceived as being careless and reckless. When asked about the change in COVID-19 concern, one participant illustrated this tension by noting, “*Others are scared while others are not; they don’t even take COVID-19 seriously*…” Similarly, another participant stated, "*People do go to bashes…*. *and other useless events…*.*”* This decrease in concern about the severity of COVID was also observed in the quantitative findings.

#### Differences in COVID-19 concern by sex & age

Similar to the differences related to fear, many participants agreed that there were sex and generational differences regarding decreased concern for COVID-19 (Table 4). When speaking to this point, many participants reiterated similar responses to those provided for fear, where, despite decreasing COVID-19 concerns, women and the elderly were perceived to still fear COVID-19. One 22-year-old participant said, *“Older people are still scared because I don’t see them lingering around like the youth*…” Another participant said, *“By looking at things*, *for me*, *it is by gender and age*. *Older females do take COVID-19 very seriously than the other gender*.*”*

#### Mistrust & fear of contact with others

Like fear of death and decreased COVID-19 concern, mistrust and fear of others illustrated how participants enacted stigma against others. Participants spoke about no longer trusting others and fearing contact with others, including family, friends, and community members. Some went as far as to accuse others of maliciously seeking to infect people with COVID-19 (Table 4). For instance, when probed about people’s unwillingness to reveal their COVID-19 infection status to others, a participant answered, “Yes, *they won’t* [reveal their COVID-19 status].” When the interviewer inquired, “*What could be the reason*?*”* The participant responded, “*They want them* [others] *to also get infected* [with COVID-19].” Frustrated about the fear of coming into contact with others and becoming infected, a participant remarked, “*We are now even afraid to go around and visit each other*. *[…] You are afraid that they might infect you with COVID-19*.”

#### COVID-19 vaccine stigma

While many participants supported COVID-19 vaccination, some anticipated being stigmatized for being vaccinated and were afraid to share their vaccination status (Table 4). The best illustration of this theme was a participant’s answer to the question of why people hide their COVID-19 vaccination status; the participant responded that “*most are scared*, *just like in my community they say that if you tell others that you have* [been] *vaccinated*, *they will bewitch you*.” To a similar question asking what makes people not want to talk about their vaccine status, a participant responded, “*Because they state that the* [COVID-19] *vaccine kills*.*”* The participant went on further, saying, *“Other men complain that it affects them when they are trying to get intimate*.” To this question, asking whether people reveal their COVID-19 vaccination status, a participant said, “*Most of them don’t say anything*.” This finding countered quantitative results, which found that almost all participants had shared their COVID-19 status with a friend or family member.

Serving as a pretext for enacting stigma against those vaccinated for COVID-19, in some cases, attitudes toward the vaccine resulted in individuals being categorized as the faithful and the faithless, with the faithless being stigmatized. Explaining how getting the COVID-19 vaccine was a testament to one’s lack of faith, to the question of how they felt about the possibility of gatherings being limited to only those who are vaccinated, a participant replied, “*They should do that because it means those who are not vaccinated have faith*…” For others, getting vaccinated went beyond observance of faith, and was tantamount to an alignment with Satan (Table 4). However, while faith played a role in the decision to get vaccinated, some believed it was a personal choice and that the outcome of that choice was ultimately “…*in the hands of God*.”

## Discussion

This mixed methods study confirmed that anticipated, perceived, and enacted COVID-19-related stigma was highly prevalent among a community of rural South African adults. However, anticipated stigma decreased between the first and second year of the pandemic. Quantitative analysis revealed that those who were less knowledgeable about COVID-19 anticipated more COVID-19-related stigma than those who were more knowledgeable. This finding was supported by qualitative results, in which some participants explicitly mentioned that having more knowledge about COVID-19 reduced their anxieties about sharing their COVID-19 status. The key quantitative findings demonstrated decreased stigma and concern about illness as the pandemic progressed. These results were further supported by the qualitative results that showed a decline in COVID-19 concern and fear of death later in the pandemic.

This community’s past experience with HIV stigma contributed to high levels of COVID-19 stigma. As a result, some individuals kept their infection and vaccination status hidden and feared others who may infect them. Concealing one’s infection status can lead to underreporting of COVID-19 infection rates. This is evident from the low estimates of previous infection rates reported by the participants in the quantitative results of this study, which are far lower than actual seroprevalence rates reported in the literature [26]. A study conducted in the study area found asymptomatic infection to be very high (85%), and given low testing availability, it is certainly possible that many individuals were unaware they had COVID-19 infection [27]. Thus, low levels of self-reported COVID-19 infection may be due to concealment, limited testing availability during this time, and high levels of asymptomatic infection, but regardless of its etiology, the connection between HIV- and COVID-19-related stigma has important implications for COVID-19 prevention and treatment programs [27]. We can leverage these findings to bolster efforts to normalize responsible disease mitigation efforts.

This study also demonstrated the role generational differences played with regard to COVID-19 concern, with more concern observed among older people. For this theme, these age-related differences are evident in both the qualitative and quantitative results. While sex differences are only demonstrated in the qualitative findings, the point estimates in the quantitative results suggest that women may potentially anticipate more stigma than men. Other studies have reported more infectious-disease-related stigma among women compared to men [28, 29], such as a South African COVID-19-related stigma study that found women experienced significantly more stigma than men [5]. This result is predictable given the higher risk of HIV among South African women compared to men [30], which may contribute to women being more concerned about COVID-19. This need for women to be more vigilant was highlighted in a 2012 study by Mashinini & Pelton-Cooper [30], where women discussed how men absolve themselves from HIV transmission by believing that only women carry HIV. This same study found that HIV prevention responsibilities largely fell on women. Similarly, the qualitative results in this current study illustrated that women wore masks and practiced other COVID-19 prevention behaviors more frequently than men. These findings highlight the pervasive unequal gender power dynamics that persist in South Africa and their interrelation with disease mitigation efforts and the need for tailored public health interventions that address both generational disparities and gender-specific concerns, thereby fostering more inclusive and effective strategies for combating infectious diseases like COVID-19.

Similar to COVID-19 concerns, quantitative and qualitative analyses revealed that anticipated stigma was more prevalent among older age groups than younger ones. Although, it should be noted that the sample size in the oldest age group was small. Nevertheless, low anticipated stigma among younger age groups is unsurprising, given the large body of evidence demonstrating that young people perceive themselves as having little to no risk of acquiring COVID-19 [9], especially early in the pandemic. Again, the qualitative themes of COVID-19 concern and fear of death demonstrated the generational differences in which the elderly were perceived to be more frightened of COVID-19 than young people and were more likely to take precautionary measures, including mask-wearing and getting vaccinated. This may be because older people are at a higher risk for COVID-19 than younger people, as well as their past experiences with HIV.

Although the quantitative results from this study found that COVID-19 knowledge was generally high among participants in 2020, those who were less knowledgeable significantly anticipated more COVID-19-related stigma. Similarly, in the qualitative results, having more COVID-19 knowledge reduced the fear of disclosing infection status to others. Also tied to knowledge was the fear of encountering stigma for getting vaccinated. Interestingly, this apprehension of not revealing one’s vaccination status in the qualitative findings was not supported by quantitative results, which found that almost all participants shared their COVID-19 vaccination status with someone. Similarly, our previous work discovered that 95.2% of participants shared their vaccination status with others [21]. However, even among those who were vaccinated, some still had concerns about the vaccine’s safety and efficacy [21]. These findings underscore the importance of health literacy and public health messaging in mitigating disease-related stigma. Health literacy can help individuals better understand and apply health information, which can, in turn, reduce fear and susceptibility to believing misinformation and stigma [31]. This approach was successfully used during the HIV epidemic, where targeted HIV/AIDS education campaigns led to diminished discrimination toward historically stigmatized communities [32].

The findings from this mixed methods study support the theoretical frameworks outlined in the literature concerning anticipated, perceived, and enacted stigma. These frameworks emphasize how the social process of stigma occurs in the context of power, wherein people emphasize differences by labeling others and assigning negative attributes or stereotypes to those labels [33]. This labeling and assigning of attributes allows those in power to create distance, separating the labeled from others in society, creating an “us” and a “them.” This process is known as “otherizing,” which allows for discrimination against the undesired labeled group [34]. The fear of being othered can lead individuals to distance themselves from this outside group. In a study of HIV risk among urban Black African women in South Africa, women believed men often engaged in HIV distancing as women proclaimed that men talked about HIV as if it belonged to other people [30]. Similarly, in this current study, distancing from COVID-19 manifested in how some participants talked about not having any personal experience with COVID-19 illness despite its ubiquitous nature.

The residents of this rural South African community greatly anticipated COVID-19-related stigma from a wide range of sources, including family, community members, and healthcare professionals. However, the marked decrease in anticipated stigma among participants a year later, as indicated by the quantitative results, supports the generalized findings that people may overestimate the extent to which others will stigmatize them. Visser et al. [35] stated that high levels of anticipated stigma might result from past stigmatizing experiences, distorting perceptions of higher levels of stigma within a community [35]. Indeed, the qualitative results from this study highlighted how the history of HIV-related stigma and discrimination in South Africa played a crucial role in how participants framed responses to questions about COVID-19-related stigma. This comparison to HIV/AIDS was common in all the themes, suggesting priming from prior HIV stigma may catalyze COVID-19-related stigma in South Africa. Not only did participants explicitly reference similarities of anticipated and enacted COVID-19-related stigma to HIV-related stigma, but they spoke of perceiving COVID-19-related stigma in the same ways as HIV-related stigma, specifically as it relates to health-seeking behaviors and the extent to which fear of stigma may prevent someone from revealing their COVID-19 infection status. Although hiding one’s COVID-19 status from others greatly parallels the HIV literature, the qualitative results demonstrate how perceptions of HIV/AIDS have changed over time. For some, HIV is now viewed as a chronic disease, is feared less than COVID-19, and, therefore, is perceived as less threatening than COVID-19. This change in perception of HIV may speak to the success of antiretroviral therapy and educational campaigns’ ability to reorder the HIV stigma landscape [36]. Similarly, as COVID-19 vaccines and treatments have become commonplace, this may also reduce stigma as individuals are less fearful of COVID-19.

Fear is an actionable driver of stigma, and someone who fears being labeled and assigned to the stigmatized group can, in turn, further exacerbate stigma [37]. Participants spoke about their fear of COVID-19 in much the same way as they spoke about their initial fear of dying from HIV/AIDS, leading to discrimination against others due to fear of death. This mistrust and fear of others translated to some participants accusing others of maliciously seeking to infect people with COVID-19. Such accusations mimic those seen during the height of South Africa’s HIV/AIDS epidemic, in which rapid HIV infection rates and increasing incidence of rape in some parts of the country were tied to the intentional infection of others [38].

The qualitative findings revealed that some people were hesitant to disclose their COVID-19 vaccine status due to fears of negative social, cultural, and religious ramifications. In some instances, the COVID-19 vaccine was associated with both death and suspicion of lacking faith in God, leading to a pervasive fear of being bewitched by others for having received the COVID-19 vaccine. This demonstrates the profound role culture and religion play in health-seeking behavior in the context of Africa. Therefore, integrating culture and religion as a central component of a health intervention may be crucial to its success.

### Strengths and limitations

This study has several notable strengths, primarily the use of mixed methods to explore COVID-19-related stigma and a large representative sample. The ability to conduct in-person follow-up IDIs with the same participants allowed for a more in-depth understanding of participants’ thoughts and feelings that the quantitative surveys could not capture. Further, at the time of the survey, there were no validated measures of COVID-19 stigma. Thus, the study was novel in adapting existing HIV stigma scales to COVID-19. The most notable strength of this study was the ability to capture changes during the pandemic through the use of repeated measures.

Even with its strengths, this study had limitations. The quantitative survey only captured anticipated stigma and did not ask about other stigma domains or intersecting stigmatized conditions (e.g., HIV, other disabilities, marginalized identities, etc.). HIV status was not collected as a variable during the time of this telephonic study because COVID-19 was the primary variable of interest, biological samples could not be collected, and asking participants about HIV may have placed participants in an uncomfortable position of disclosing their status in the presence of household members. Further, the unvalidated COVID-19-related stigma measures used in this study may be considered a limitation; however, Cronbach’s alpha values for this survey were promising. In the qualitative component of the study, IDIs conducted in participants’ homes made controlling for distractions and other external factors difficult. Other family members would sometimes interject, possibly leading to inauthentic participant responses for sensitive topics, perhaps biasing some results. Similarly, as the interviewers were from the local area, this may have influenced the participants’ responses due to the close-knit nature of the rural community. Lastly, translating interviews into English may have lost nuances of some ideas and thoughts.

## Conclusion & recommendations

This study found elevated levels of COVID-19-related stigma in a representative sample of rural South African adults. Stigma was especially high among women, older adults, and those who were less knowledgeable about COVID-19. Given this evidence, South African governmental bodies should prioritize efforts to mitigate the social stigma associated with COVID-19, especially in rural areas, to bolster public health efforts combating COVID-19. We acknowledge that the threat of COVID-19 and its adverse effects globally have significantly decreased. However, we can learn a lot about various types of COVID-19-related stigma that can be useful in preparing for future infectious disease outbreaks. Based on the evidence presented in this study, it is important for South African government authorities to quickly prioritize efforts to reduce social stigma surrounding infectious diseases, particularly in rural areas. Doing so will help mitigate the spread of infectious diseases, allowing for the better implementation of effective intervention measures. We recommend employing environmental and structural interventions [39] that address actionable drivers of stigma, such as those highlighted by Nyblade and colleagues [37], who call attention to three interconnected principles for HIV-related stigma reduction that also prove helpful in the context of COVID-19 and any other future infectious disease outbreaks. These include prompt action in creating awareness of stigma, addressing fears and misconceptions, and understanding, recognizing, and acknowledging negative attitudes toward stigmatized groups. Applying these principles, especially in rural South African communities, is essential for reducing COVID-19-related stigma, which will strengthen COVID-19 mitigation and intervention measures and could be used as a template for addressing other forms of infectious disease-related stigma.

## Supporting information

S1 File COREQ checklist. (PDF)

S2 File Inclusivity form. (DOCX)
